# Aramid Pulp Reinforced Clay Aerogel Composites: Mechanical, Thermal and Combustion Behavior

**DOI:** 10.3390/gels8100654

**Published:** 2022-10-14

**Authors:** Xiaowu Wang, Yang Wang, Mengtian Sun, Guichao Wang, Qiong Liu, Ming Li, Yury M. Shulga, Zhi Li

**Affiliations:** 1 School of Resource and Safety Engineering, Central South University, Changsha 410083, China; 2 Institute of Problems of Chemical Physics, Russian Academy of Sciences, 142432 Chernogolovka, Russia; 3 National University of Science and Technology MISIS, Leninsky pr. 4, 119049 Moscow, Russia

**Keywords:** aramid pulps, clay aerogel, mechanical properties, thermal properties, combustion behavior

## Abstract

In this work, we reported that aramid pulps (AP) reinforced clay aerogel composites with improved mechanical strength, good thermal insulation and fire resistance based on the combination of AP, Poly(vinyl alcohol) (PVA) and sodium montmorillonite (MMT), which present a promising prospect in the thermal insulation application. The PVA-MMT-AP_x_ (x: denotes the mass content of AP) aerogel composites present an isotropic “lamella-honeycomb” porous structure, which endows them with excellent comprehensive performance. With the AP content increasing, the extremely low density is kept, ranging between 67–73 mg/cm^3^, and the low thermal conductivity is maintained within 40.9–47.9 mW·m^−1^·K^−1^. The mechanical strength is significantly improved with the maximum compressive modulus increasing from 2.95 to 5.96 MPa and the specific modulus rising from 44.03 to 81.64 MPa∙cm^3^/g. Their detailed heat transfer process has been analyzed, which provides a deep understanding to the low thermal conductivity of the PVA-MMT-AP_x_ aerogel composites. Based on the combination of thermogravimetric analysis and combustion behavior, the PVA-MMT-AP_x_ aerogel composites are demonstrated to possess improved thermal stability and fire resistance. This study puts forward a facile approach to utilizing AP to reinforce clay aerogel composites, which provides new insight into the development of thermal-insulating, fire-safe and high-strength thermal insulation materials.

## 1. Introduction

Clay aerogels are ideal candidates for thermal insulation materials with low density, low thermal conductivity and good fire resistance, and recently have aroused much interest due to their cost-effective and eco-friendly freeze-drying process [1,2,3,4,5,6]. However, pure clay aerogels are fragile and low-strength to satisfy the practical applications. For instance, the compressive modulus of the freeze-dried 5 wt% clay suspension is less than 10 kPa [7]. To improve the mechanical properties of clay aerogels, incorporating polymers has been confirmed as a reliable method and various polymers have been widely used, such as poly(vinyl alcohol) (PVA) [8], polyimide [9], natural rubber [10], pectin [11], casein [12], starch [2] and alginate [13]. Thereinto, PVA as a high-molecular polymer tends to show excellent toughening effect, whether in the clay aerogel, silica aerogel [14] or the phase change material aerogel [15]. These clay/polymer aerogels usually show higher mechanical strength and are regarded as the desirable alternative to commercial insulation foams.

In spite of this, further enhancement of mechanical properties of clay aerogel composites will broaden the range of their applications. A high polymer content usually leads to the deterioration of the thermal insulation as well as flame retardancy, though it does increase the mechanical properties [16]. Several studies have confirmed that incorporating biologically-based fibers can further increase the mechanical properties of clay aerogel composites, and the effect of fiber diameter and length on mechanical properties have been investigated [17,18,19]. This fiber-incorporating strategy without extra chemical crosslinking could be a good choice from a maneuverable point of view [20,21]. However, little attention has been paid to the comprehensive properties, particularly thermal and combustion properties, when further toughening the clay aerogel composites.

According to the current investigations, reinforcing fibers with thinner diameter, higher specific surface and better flexibility will do a greater favor to retain the composite matrix intact. Besides, lower density and shorter length of fibers make them evenly disperse into solvent and avoid sedimentation [22]. Aramid pulps (AP) are highly fibrillated aramid fibers and possess the excellent physicochemical properties such as low density, high toughness, good flexibility, high thermal stability and self-extinguishment [23,24,25,26,27]. Recent reports have indicated that AP as reinforcement showed good compatibility with polymer, which is very suitable for a clay/polymer system [28,29,30]. From these points of view, AP can be a good candidate for achieving our research purpose. Note that it is the first appearance of organic AP applied in a clay aerogel material.

Here, we utilize AP to reinforce clay aerogel aiming to achieve low density, excellent thermal insulation as well as flame retardancy. The mechanical, thermal and combustion behavior of the AP-reinforced clay aerogel composites are characterized and investigated systematically. This study confirms the availability of using AP as reinforcement for clay aerogels and develops a new fire-safe and efficient thermal insulation material, which renders a new insight into fabricating clay aerogel composites with excellent comprehensive performances.

## 2. Results and Discussion

### 2.1. Basic Physicochemical Characterization

**Figure 1:**
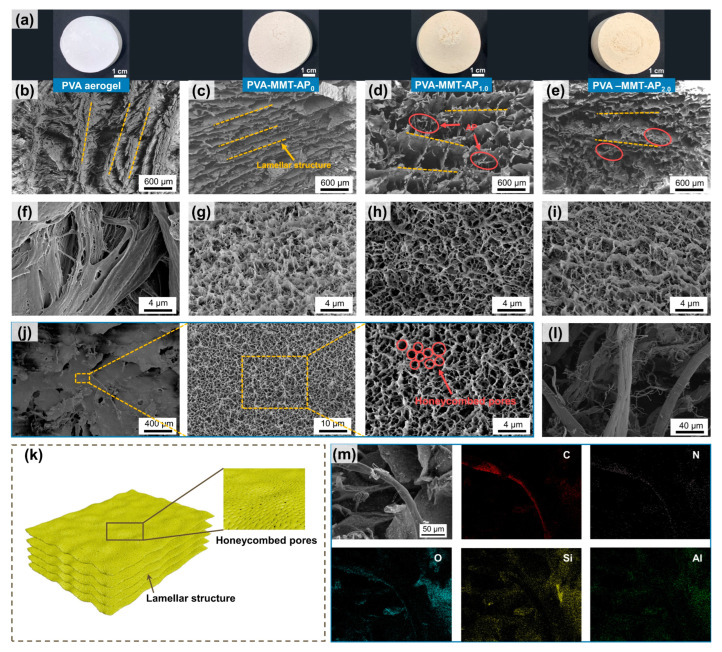
(**a**) The prepared samples from left to right are the PVA aerogel, PVA-MMT-AP_0_, PVA-MMT-AP_1.0_ and PVA-MMT-AP_2.0_, respectively. The microstructures of the (**b**,**f**) PVA aerogel, (**c**,**g**) PVA-MMT-AP_0_, (**d**,**h**) PVA-MMT-AP_1.0_ and (**e**,**i**) PVA-MMT-AP_2.0_, respectively. (**j**) The lamella of PVA-MMT-AP_1.0_ and (**k**) the schematic illustration of the “lamella-honeycomb” porous network structure. (**l**) AP. (**m**) The element distributions of the PVA-MMT-AP_1.0_.

As shown in Figure 1a, the PVA aerogels and PVA-MMT-AP_x_ present a regular and intact cylinder, and their colors change from white to yellowish with the content of MMT and AP increasing. Although all the samples have volume shrinkage, the radial shrinkage of PVA-MMT-AP_x_ (1.8~3.2%) is significantly smaller than that of the PVA aerogel (8.7%); and as a consequence, the bulk densities of the PVA-MMT-AP_x_ are far lower than that of the PVA aerogel (Table S1). In Figure 1b–e, it finds that the PVA aerogel and PVA-MMT-AP_x_ have a typical layered structure (labeled as dotted lines), in which the micron-sized AP (labeled as ellipses) are embedded in the aerogel matrix, acting as reinforcement. The finer microstructure in Figure 1f–j reveals that honeycombed pores distribute all over the lamellas of PVA-MMT-AP_x_, while the PVA aerogel shows thicker lamellas and fewer pores on the lamellas.

Note that the microstructure of materials fabricated by a freeze-drying method is directly depended on the growth of ice crystals. During the freezing process, the ice crystals grow along the temperature gradient and squeeze the matrix aside into the ice grain boundaries, inducing parallel alignment of the matrix [31]. For the PVA aerogel, a high-viscosity precursor solution inflicts great resistance on the growth of ice crystals, resulting in smaller and fewer ice crystals. For the PVA-MMT-AP_x_, the lower content of polymer presents little resistance, which is conducive to the growth of ice crystals. As a consequence, the lamellas with various compactness are formed finally for the PVA aerogel and PVA-MMT-AP_x_.

The “lamella-honeycomb” porous layered structure (Figure 1k) imparts multifunctional potentials to the as-prepared clay aerogel composites, e.g., adsorption and carrier materials. This kind of structure is the coupling of the layered structure (discussed above) and honeycomb structure. For the formation of the honeycomb structure, the hydrophilic hydroxyls from the PVA and MMT bind with neighboring water molecules to generate the bound water, which is harder to crystallize before the freeze of free water. Hence, the formed honeycombed pores on the lamellas may be caused by the fractal growth of the smaller ice crystals derived from this bound water [11,32]. Furthermore, the element distributions tested by the SEM-EDS mapping further confirm the independent existence of micron-sized AP (C, N elements) and MMT (Si, Al elements) (Figure 1m).

**Figure 2:**
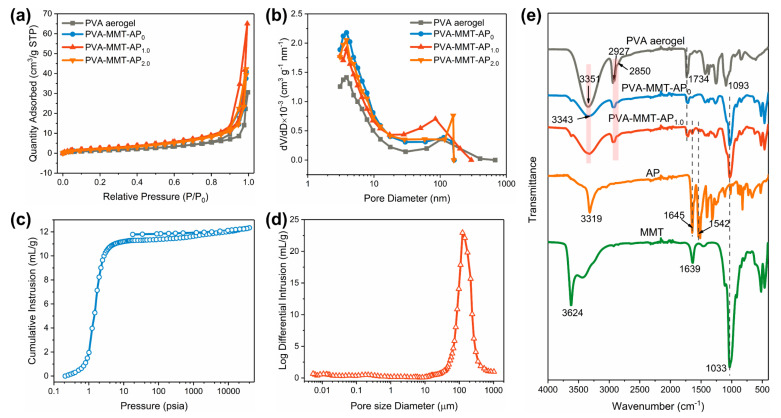
(**a**) N_2_ adsorption–desorption isotherms and (**b**) pore size distribution of PVA aerogel, PVA-MMT-AP_0_, PVA-MMT-AP_1.0_ and PVA-MMT-AP_2.0_. (**c**) Mercury intrusion–extrusion isotherms and (**d**) pore size distribution of PVA-MMT-AP_1.0_. (**e**) FTIR spectra of AP, MMT, PVA aerogel, PVA-MMT-AP_0_ and PVA-MMT-AP_1.0_.

Figure 2 characterizes the pore structures and surface chemical groups of the clay aerogel composites. All the curves follow the IV type isotherm with the H3 hysteresis loop, and the most probable pore sizes concentrate at ~4 nm and ~100 nm, respectively, which confirms the presence of mesopores and macropores in the clay aerogel composites [33]. As discussed in Figure 1, the clay aerogel composites have abundant porous structures, especially a lot of micron-sized pores. Due to the limitation of nitrogen sorption analysis [34], these large pores cannot be included in the measured results. That is the reason why all the nitrogen adsorbed quantity, pore volume (*V_pore_*) and specific surface area (*S_BET_*) (Table S2) are so low. Besides, the nitrogen sorption also leads to a huge discrepancy between the *V_pore_* and *V_total_* in Table S1, of which *V_pore_* is calculated from the nitrogen sorption and *V_total_* is calculated as per the formula, *V_total_* = (1/*ρ_b_* − 1/*ρ_s_*), (*ρ_b_*, *ρ_s_* are the bulk density and skeletal density).

For further understanding the pore structure, the mercury intrusion porosimeter is employed, and here we take PVA-MMT-AP_1.0_ as an example. As shown in Figure 2c, the intrusion volume increases slowly below 1 psia, then abruptly increases until reaching a nearly stable state, around 10 psia, because the external pressure is inversely proportional to pore size, i.e., more pressure is needed to press mercury into smaller pores. Figure 2d shows that the pore size primarily ranges between 50 μm and 500 μm and the most probable pore size locates at 100 μm. These pore characteristics further confirm the presence of abundant micron-sized pores in the PVA-MMT-AP_x_. Via the mercury intrusion porosimeter, the specific surface and the pore volume for PVA-MMT-AP_1.0_ are tested as 157.15 m^2^/g and 12.33 cm^3^/g, respectively (Table S3), which are more reasonable and closer to the theoretical values, e.g., pore volume. It also indicates the mercury intrusion porosimeter is more appropriate for characterizing the pore structures of the prepared clay aerogel composites.

The FTIR spectra are recorded in Figure 2e. For the PVA aerogel, the peak at 3351 cm^−1^ corresponds to the stretching vibration of -OH groups, while the absorption bands at around 2927 cm^−1^ and 2850 cm^−1^ are assigned to the asymmetric and symmetric stretching vibration of -CH_2_ groups, respectively. The characteristic peaks at 1734 cm^−1^ and 1093 cm^−1^ are ascribed to the stretching vibration of C=O and C-O bonds, respectively [35,36]. To the pure AP, the typical characteristic peaks located at 3319 cm^−1^, 1645 cm^−1^ and 1542 cm^−1^ correspond to the stretching vibration of the N-H, C=O and the bending vibration of N-H, respectively [23,25,37]. In regard to the MMT, the peak at 3624 cm^−1^ arises from the stretching vibration of single ‘inner’ hydroxyl bonded to octahedral cations [38]. It is important that this peak is retained in the PVA-MMT-AP_0_ and PVA-MMT-AP_1.0_, which is evidence of the preservation of the internal structure in the clay particles of the aerogel composites. The peaks at 1639 cm^−1^ and 1033 cm^−1^ are attributed to the stretching vibration mode of absorbed water and Si-O bonds, respectively [5,39]. Compared to the PVA aerogel and MMT, a red shift towards 3343 cm^−1^ in the stretching vibration of hydroxyls for PVA-MMT-AP_0_ is observed, which should be ascribed to the formation of hydrogen bonds between the MMT and PVA [37,40]. Furthermore, there is no other new absorption peak in the spectra of PVA-MMT-AP_1.0_, indicating that only physical combination occurs among the AP, PVA and MMT.

### 2.2. Mechanical Properties

**Figure 3:**
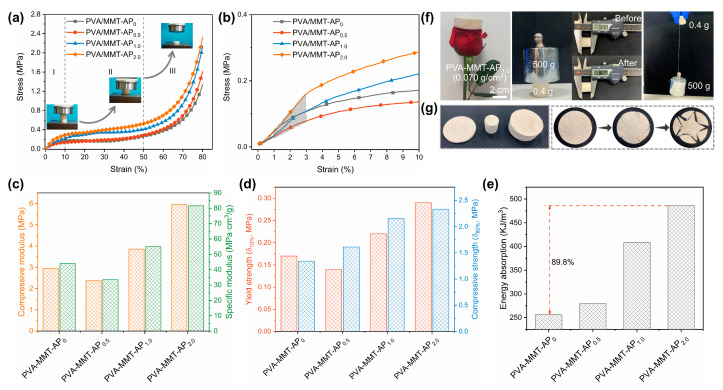
(**a**) Compressive stress-strain (δ-ε) curves and (**b**) the enlarged δ-ε curves of the PVA-MMT-AP_x_ aerogel composites (insets: photos of the PVA-MMT-AP_x_ under the compression test). The linear gray region (1% < ε < 3%) is used to determine the compressive modulus. (**c**) Compressive modulus and specific modulus. (**d**) Yield strength (δ_10%_) and compressive strength (δ_80%_). (**e**) Absorption energy (at 80% strain) of the PVA-MMT-AP_x_. (**f**) The PVA-MMT-AP_x_ standing on the rose; pressed or pulled by a weight of 500 g. (**g**) The clay aerogel composites with customizable shapes and their machinability.

Figure 3a,b shows the compressive stress-strain curves of PVA-MMT-AP_x_. As shown in the insets, the specimens exhibit typical compression behavior of foam materials, which can be mainly divided into three stages. (I) The linear elastic stage ranges between a low strain region of 0 < ε < 10%, reflecting the elastic deformation of the skeletons of the clay aerogel composites. (II) The yield stage within the strain of 10% < ε < 50% shows that the stress increases slowly with the strain, and the yielding plateau also appears at this stage due to the collapse of pore structures. (III) The densification stage ranges within a higher strain of ε > 50%, during which the stress increases sharply and the specimens are compacted completely [35,41].

In Figure 3c, with the addition of AP increasing, the compressive modulus and specific modulus of the aerogel composites have been significantly enhanced, from 2.95 MPa to 5.96 MPa and 44.03 MPa∙cm^3^/g to 81.64 MPa∙cm^3^/g, respectively. The yield strength (δ_10%_) and compressive strength (δ_80%_) in Figure 3d increase up to 0.29 MPa and 2.33 MPa, respectively. Moreover, the improvement of compressive strength is significant, though the low AP content has a limited influence on the compressive modulus and yield strength. In Figure 3e, it also finds that the energy absorption of the PVA-MMT-AP_2.0_ increases 89.8% compared to that of the PVA-MMT-AP_0_, indicating a potential for acting as packing materials. Figure 3f shows that the lightweight PVA-MMT-AP_x_ aerogel can be easily supported by a rose and also can withstand the compression and pull without obvious deformation under an external force of 1250 times its weight. Furthermore, the as-prepared clay aerogel composites have good machinability, which enables them to be carved to desired shapes without any crack and powder in Figure 3g. All the discussions above suggest that the clay aerogel composites possess good mechanical properties which renders them with the capacity to satisfy the requirement of given application scenarios.

### 2.3. Thermal Insulation Properties

**Figure 4:**
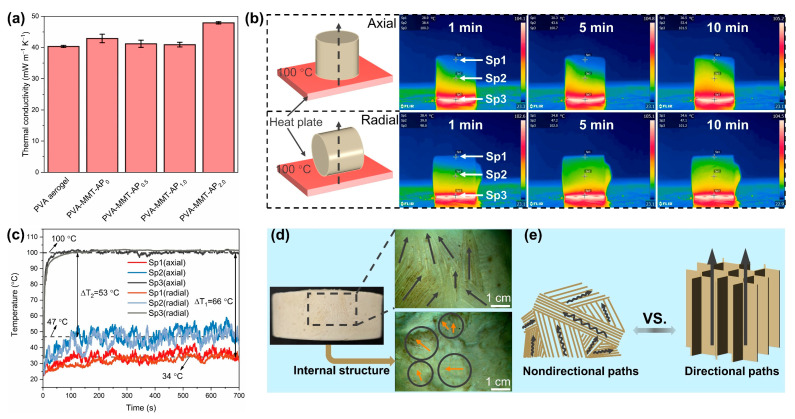
(**a**) Thermal conductivity of the PVA aerogel and PVA-MMT-AP_x_ aerogel composites. (**b**) Infrared thermographic images of the PVA-MMT-AP_1.0_ with a size of Φ2 cm × 2 cm on the hot surface of 100 °C. (**c**) The time-dependent temperature profile at the points of Sp1, Sp2 and Sp3 in the axial and radial directions (Sp1, on the upper end; Sp2, on the middle; and Sp3, on the bottom). (**d**) The texture of the PVA-MMT-AP_1.0_. (**e**) The schematic of the heat transfer through nondirectional or directional paths.

The investigation of thermal transport properties is of significant interest for thermal insulation materials. As shown in Figure 4a, the as-prepared aerogel composites display a favorable thermal conductivity of 40.3–47.9 mW·m^−1^·K^−^^1^, which are comparable to the traditional thermal insulation materials, such as commercial polystyrene and polyurethane foams (30–50 mW·m^−1^·K^−1^) [42]. At a lower AP content (<2.0 g), the PVA-MMT-AP_x_ still retains lower thermal conductivity and the PVA-MMT-AP_1.0_ exhibits the lowest thermal conductivity of 40.9 mW·m^−1^·K^−1^. At a larger AP content (≥2.0 g), the thermal conductivity has a slight increase, because excessive AP provide more heat transfer paths.

The infrared thermographic images of the PVA-MMT-AP_1.0_ have been recorded for visually evaluating its thermal insulation performance (Figure 4b). It can be observed that the upper and middle of the PVA-MMT-AP_1.0_ maintain a lower surface temperature at 1 min, 5 min and even 10 min, respectively, showing excellent thermal insulation in both the axial and radial directions. The time-dependent temperature profiles of the three tested points (Sp1, Sp2 and Sp3) in the axial and radial directions are recorded in Figure 4c. It finds that the temperatures of the three tested points increase with the heating time, and then reach equilibrium. The ∆T_1_ (between Sp1 and the hot surface) and ∆T_2_ (between Sp2 and the hot surface) are finally kept at about 66 °C and 53 °C, respectively. In addition, the surface temperature profiles of the three points present similarly in the axial and radial directions, indicating the equally excellent thermal insulation of the aerogel composites in both the axial and radial directions.

As far as known, the directional structure will impair the thermal insulation performance in a specific direction, because more heat chooses to transfer uninterruptedly along the directional paths [1,43,44]. For the prepared aerogel composites, heat transfer is inhibited in both the axial and radial directions due to the isotropic structure comprised of the nondirectional tortuous paths (Figure 4d,e), and the aerogel composites thus show good thermal insulation performance in both directions. Predictably, this characteristic plays an important role in practical thermal insulation applications.

**Figure 5:**
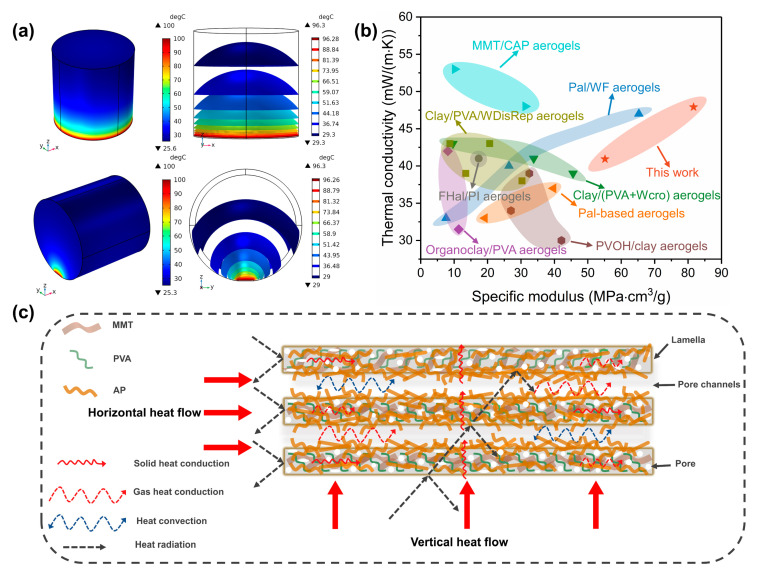
(**a**) The temperature profiles and isothermal surfaces (at 500 s) in the axial and radial directions. The heat transfer of a cylinder sample with a size of Φ2 cm × 2 cm on a hot surface of 100 °C is simulated using the COMSOL Multiphysics software and all the modelling parameters are kept consistent with the infrared thermal imaging experiment. (**b**) The thermal conductivity versus specific modulus of the PVA-MMT-AP_x_ and other clay aerogel composites [5,39,41,45,46,47,48,49]. (**c**) The schematic of the heat transfer of the aerogel composites in two different directions.

Here, the transient heat transfer is conducted within a total calculation time of 500 s (see Video S1 and S2). More details about the simulation are presented in Figure S1 in the Supplementary Materials. The temperature profiles show that the lowest surface temperatures in the axial and radial directions are 25.6 °C and 25.3 °C, respectively, which further confirms the excellent thermal insulating performance of the aerogel composites (Figure 5a). Furthermore, the temperature profiles in the aerogel composite are theoretically demonstrated to be upward convex surfaces because of air convection, and this offers a guide for designing the thermal management system.

Figure 5b compares the thermal conductivity and specific modulus of the PVA-MMT-AP_x_ and other clay aerogel composites. It finds that the PVA-MMT-AP_x_ exhibits much higher specific modulus among these ever-reported aerogel composites; meanwhile, their thermal conductivities remain close to those of the most clay aerogel composites. Considering these two points, the nice mechanical property and satisfactory thermal conductivity indicate the PVA-MMT-AP_x_ is an excellent thermal insulation material.

Figure 5c illustrated the heat transfer of the PVA-MMT-AP_x_. Generally, the effective thermal conductivity consists of the contributions of convection (*λ_c_*), conduction (where *λ_g_* represents gas conduction and *λ_s_* represents solid conduction) and radiation (*λ_r_*) [44]. *λ_c_* and *λ_r_* become negligible for the aerogel composites due to the small pore size (less than 1 mm) and the relatively lower ambient temperature [50,51].

The gas conduction *λ_g_* is strongly dependent on the porosity and pore size of materials and can be calculated by Kaganer’s model [52] as per Equation:

(1) $$\lambda_{g}=\frac{\lambda_{g0}\Pi }{1+2\beta K_{n}}$$

where *λ_g_*_0_ stands for the gaseous thermal conductivity of air in free space, 26 mW·m^−1^·K^−1^; Π is the porosity, *β* is a coefficient dependent on the energy accommodation coefficient and adiabatic coefficient of gas. For air in aerogel, *β* ≈ 2. *K_n_* is the Knudsen number which can be estimated as Equation:

(2) $$K_{n}=\frac{l_{m}}{\delta }$$

where *l_m_* represents the mean free path of a gas molecule, 73 nm, and *δ* is the characteristic system size, i.e., the average pore size of the porous aerogel composites.

The solid conduction *λ_s_* can be estimated by a weighted average of the effective solid conduction values $\lambda_{sol}^{*}$ of the individual components of the aerogel composite by using Equation [44,53]:

(3) $$\lambda_{sol}^{*}=\frac{\lambda_{sol}}{1+\lambda_{sol}\frac{R_{k}}{d}}$$

where *λ_sol_* is the solid thermal conductivity of the individual components of the aerogel composite, *R_k_* is the so-called Kapitza resistance and *d* is the particle size.

For the PVA-MMT-AP_1.0_, the *λ_g_* is estimated to be approximately 12.88 mW·m^−1^·K^−1^. The *λ_s_* of the aerogel composite is effectively reduced from ~91 mW·m^−1^·K^−1^ for an equivalent bulk material to ~43 mW·m^−1^·K^−^^1^ for the as-prepared aerogel composites due to the phonon scattering effects (see Discussion S1 and S2 and Table S4). Note that estimating the effective thermal conductivity is knotty, and the simple linear superposition of convection, conduction and radiation has been confirmed to be imprecise, and extra factors must be considered, such as the solid-gas coupling effects. Moreover, the structural characteristics of a material should be taken into account for an accurate numerical simulation [54,55].

### 2.4. Thermal Stability and Combustion Behavior

**Figure 6:**
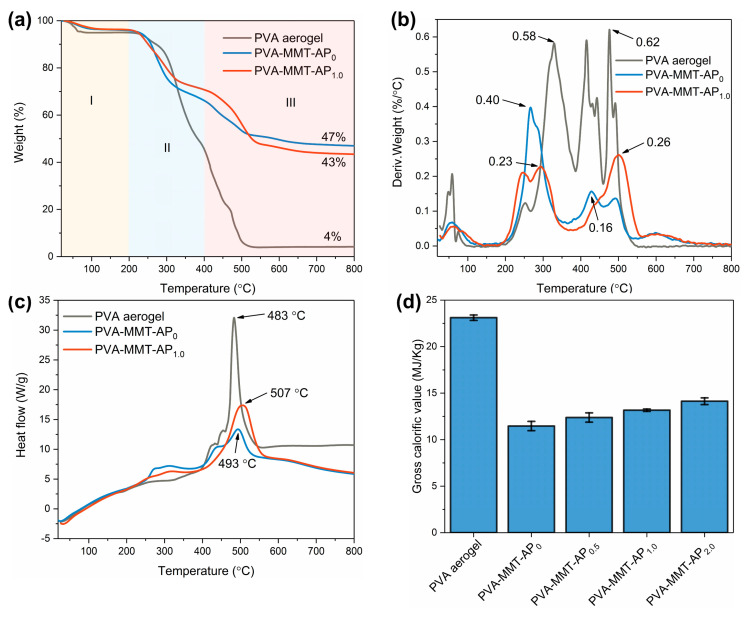
(**a**) TG, (**b**) DTG and (**c**) DSC curves of the pure PVA aerogel, PVA-MMT-AP_0_ and PVA-MMT-AP_1.0_. (**d**) The gross calorific values of the pure PVA aerogel and PVA-MMT-AP_x_.

Figure 6a–c shows the thermal analyses of the pure PVA aerogel and PVA-MMT-AP_x_ and the detailed thermal decomposition data have been listed in Table 1. According to the TG curves in Figure 6a, the thermal decomposition of the pure PVA aerogel and PVA-MMT-AP_x_ are divided into three stages. At Stage I, the slight weight losses below 200 °C are attributed to the evaporation of the adsorbed water as well as the crystalline water from MMT [45]. In Stage II, the second weight losses that occur at 200–400 °C are mainly ascribed to the decomposition of hydroxyls in PVA [14,56]. At Stage III, the weight losses observed at 400–600 °C are assigned to the thermal decomposition of the PVA backbones and AP [25,57].

The DTG curves are presented in Figure 6b and the maximum decomposition rates are labeled. In Table 1, it finds that the temperatures at the maximum decomposition rates (T_max_) of the PVA-MMT-AP_1.0_ increase 20 °C and 65 °C at the Stage II and Stage III, respectively, when compared to that of the PVA-MMT-AP_0_. This increasement should be ascribed to the introduction of AP. Furthermore, the temperature at the exothermic peak for the PVA-MMT-AP_1.0_ is slightly improved to 507 °C compared to those of the pure PVA aerogel and PVA-MMT-AP_0_ in Figure 6c. All these results confirm that the addition of AP is beneficial to the thermal stability of the aerogel composites to some extent. Figure 6d shows that the PVA-MMT-AP_x_ has gross calorific values (GCV) ranging between 11.46–14.13 MJ/kg, which are significantly lower than that of the PVA aerogel. Considering their thermal insulation applications, these lower GCV benefit to the thermal safety of the clay aerogel composites.

**Figure 7:**
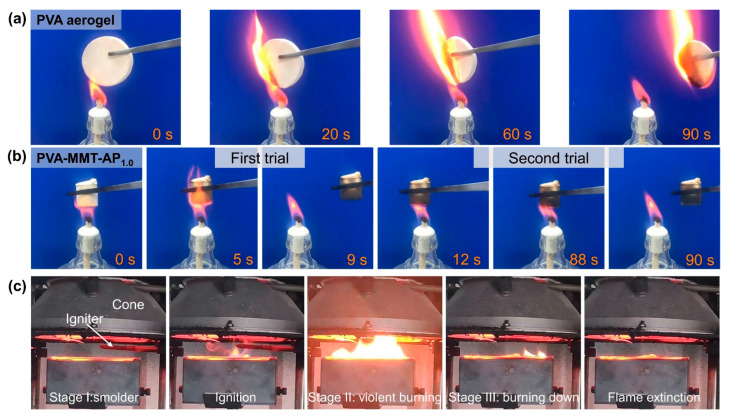
The combustion behavior of (**a**) the PVA aerogel and (**b**) PVA-MMT-AP_1.0_ under an alcohol lamp. (**c**) The combustion process of the PVA-MMT-AP_1.0_ under a heat flux of 50 kW/m^2^.

As shown in Figure 7a, the PVA aerogel can be easily ignited and keeps burning until exhausted after withdrawing the ignition source. After continuously igniting for about 80 s, the PVA-MMT-AP_1.0_ is still hard to be ignited with exhibiting self-extinguishment in Figure 7b. From this view, the clay aerogel composites have better fire resistance. Figure 7c shows the combustion process of the PVA-MMT-AP_1.0_ can be divided into three stages. (I) Smolder: the PVA-MMT-AP_1.0_ begins to decompose and releases flammable volatiles and smoke when exposed to the radiative heat source. (II) Violent burning: the orange flames grow rapidly on the PVA-MMT-AP_1.0_ once ignited by the igniter. (III) Burning down: the flames gradually split into several small flames and extinguish finally.

**Figure 8:**
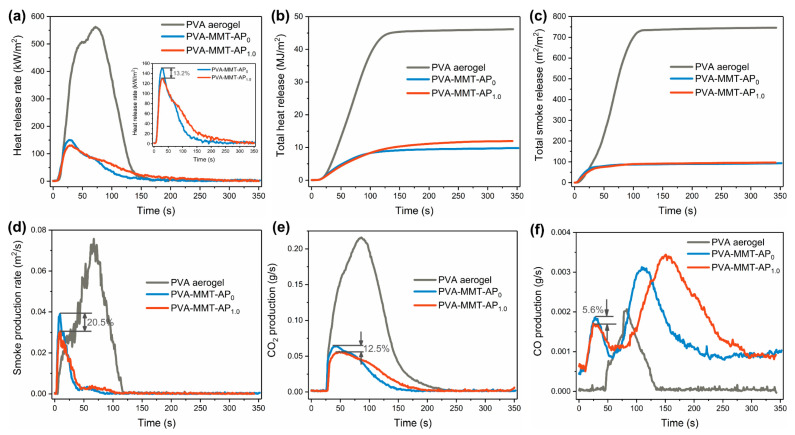
The (**a**) heat release rate (HRR), (**b**) total heat release (THR), (**c**) total smoke release (TSR), (**d**) smoke production rate (SPR), (**e**) CO_2_ production and (**f**) CO production of the PVA aerogel, PVA-MMT-AP_0_ and PVA-MMT-AP_1.0_ under a heat flux of 50 KW/m^2^.

The combustion characteristics by a cone calorimeter are shown in Figure 8, and the time to ignition (TTI), peak of heat release (PHRR), time to PHRR (TTPHRR), total heat release, fire growth rate (FIGRA, defined as the ratio of PHRR to TTPHRR), total smoke release and residue are listed in Table 2. All the aerogel composites have a lower TTI of ~2 s, indicating that these aerogel composites easily catch fire under this condition. In Figure 8a,b, the PHRR and THR of the PVA aerogel reach as high as 562.8 kW/m^2^ and 46.2 MJ/m^2^, respectively, and almost no residue is observed, which suggests that the PVA aerogel has a very high fire risk. Compared to the PVA aerogel, the PVA-MMT-AP_0_ and PVA-MMT-AP_1.0_ display the significantly decreased PHRR and THR. Furthermore, the FIGRA of the PVA-MMT-AP_0_ and PVA-MMT-AP_1.0_ are reduced and their residues are up to about 40%. The decreased PHRR, THR and FIGRA as well as increased residues indicate that the fire risk of the PVA-MMT-AP_x_ significantly decreases. Compared to the PVA-MMT-AP_0_, the PHRR of the PVA-MMT-AP_1.0_ has a drop of 13.2% and the FIGRA slightly decreases from 5.6 kW/m^2^/s to 4.7 kW/m^2^/s. Note the addition of AP slightly increases the THR of the aerogel composites, because these organic AP would also release heat, and this point is also verified by the increasement of the GCV discussed above.

In regard of smoke, both of the PVA-MMT-AP_0_ and PVA-MMT-AP_1.0_ release less smoke with the TSR decreasing from 746.5 m^2^/m^2^ (the PVA aerogel) to 93.4 m^2^/m^2^ (Figure 8c). Meanwhile, the SPR and CO_2_ production also decrease obviously in Figure 8d,e. For the CO production, the PVA-MMT-AP_0_ and PVA-MMT-AP_1.0_ have two distinct peaks (Figure 8f). The first one should be attributed to the incomplete combustion of the flammable volatiles, while the second peak is ascribed to the existence of smolder at the stage of burning down. Considering the combustion process of the aerogel composites, the characteristics of the CO production should depend on the first peak (at the violent burning stage) instead of the second one (at the burning down stage). Furthermore, compared to those of the PVA-MMT-AP_0_, it further finds that the peaks of the SPR, CO_2_ and CO productions of the PVA-MMT-AP_1.0_ are reduced by 20.5%, 12.5% and 5.6%, respectively. Similarly, the addition of AP also slightly increases the TSR of the PVA-MMT-AP_1.0_.

In general, the fire hazards of the PVA-MMT-AP_x_ are significantly decreased compared to that of the PVA aerogel. Between the PVA-MMT-AP_0_ and PVA-MMT-AP_1.0_, the introduction of AP decreases the PHRR and FIGRA, and improves the thermal stability, all of which benefits reducing the fire hazards to some extent.

## 3. Conclusions

Herein, lightweight and mechanically strong clay aerogel composites with low thermal conductivity and fire hazards were facilely fabricated by using AP as reinforcement. The physical combination is confirmed between the AP and clay aerogel matrix, and the firmly embedded micron-sized AP results in significantly increased mechanical strength and energy absorption performance. The constructed “lamella-honeycomb” porous structure contributes to the low thermal conductivity, and the isotropic structure guarantees the excellent thermal insulation performance in both the axial and radial directions. Furthermore, it demonstrates that the introduction of AP improves the thermal stability and reduces the fire hazards, which is beneficial to practical thermal insulation applications. This work validates the feasibility of using AP to reinforce clay aerogel composites, and provides an engineering example to develop a fire-safe and high-strength material for practical thermal insulation applications.

## 4. Experimental Section

### 4.1. Materials

Poly(vinyl alcohol) (PVA, polymerization degree of 1700, alcoholysis degree of 88%) was purchased from Aladdin Reagent Co., Ltd., Shanghai, China. Clay as a sodium montmorillonite (Na^+^-MMT, PGW grade, cation exchange capacity (CEC) 145 mequiv/100 g) was provided by Nanocor Inc., Chicago, IL, USA. Aramid pulps (AP) with an average length of 1 mm and width of 11 μm were supplied by Cangzhou Zhongli New Material Technology Co., Ltd., Cangzhou, China. Deionized water was used in the whole process of the experiments and prepared by a laboratory water purification system (Eco-S15UVFV, HHitech, Shanghai, China). All chemicals were used as received without further purification.

### 4.2. Preparation of AP Reinforced Clay Aerogel Composites

**Figure 9:**
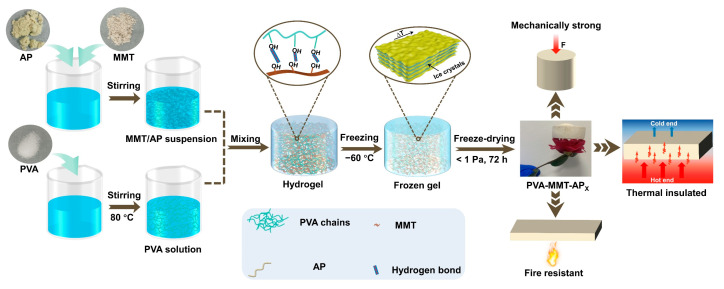
Schematic illustration for the preparation of PVA-MMT-APx aerogels composites.

A facile mixing–molding–freezing procedure followed by vacuum drying was employed in this work (Figure 9). MMT/AP suspension and PVA solution were fully mixed to build a hydrogel, during which hydrogen bonds were formed between PVA chains and MMT. After vacuum drying, AP-reinforced clay aerogel composites with desirable thermal insulation and flame retardancy were fabricated. The details of the preparation process are described as follows.

A desired amount of AP and 5 g MMT were evenly dispersed in 95 mL deionized water under vigorous stirring to form a 5 wt% MMT/AP suspension. Concurrently, 10 wt% PVA solution was prepared in deionized water at 80 °C. Then 50 g of 10 wt% PVA solution and the mentioned MMT/AP suspension were mixed well to create a uniform hydrogel, which was subsequently poured into a mold and frozen in a cold trap (−60 °C). The frozen gels were then dried using a vacuum freeze-dryer (SCIENTZ-12N, Ningbo SCIENTZ Biotechnology Co., Ltd., Ningbo, China) under less than 1 Pa for 72 h and finally the aerogels were obtained. The samples were labeled as PVA-MMT-AP_x_ (x = 0, 0.5, 1.0, 2.0), where x represents the mass of AP. In addition, the pure PVA aerogel was fabricated with 10 wt% PVA solution following the same procedure.

### 4.3. Characterization

The bulk density (*ρ_b_*) was calculated by the formula *ρ_b_* = *m*/*v*, where *m* and *v* stand for the mass and volume of the samples, respectively. The radial shrinkage (*S*) can be obtained through the formula *S*(%) = 100% × (*d*_0_ − *d*)/*d*_0_, where *d*_0_ and *d* represent the diameter of frozen gel and the final sample, respectively. The porosity and the skeletal density were calculated by the following Equations:

(4) $$Porosity\%=\left(1-\frac{\rho_{b}}{\rho_{s}}\right)\times 100\%$$

(5) $$\frac{1}{\rho_{s}}=\sum_{i=1}^{n}\frac{w_{i}}{\rho_{i}}$$

where *ρ_s_* is the skeletal density of aerogel composites, *w_i_* and *ρ_i_* are the weight fraction and skeletal density of each component, respectively. The skeletal density of MMT, PVA and AP are 2.6 g/cm^3^, 1.3 g/cm^3^ and 1.45 g/cm^3^, respectively [22].

The microstructure was observed by scanning electron microscopy (SEM, Zeiss Sigma 300, Oberkochen, Germany) at an accelerating voltage of 3 kV. The samples were pasted on the bench through the conductive tape and coated with gold on the surface before observation. Nitrogen adsorption–desorption isotherms were measured at 77 K with an automatic surface area and porosity analyzer (AUTOSORB IQ, Quantachrome, FL, USA). The mercury-injection test was carried out by an automatic mercury porosimeter (Micromeritics instrument AutoPore IV 9510, Atlanta, GA, USA). The surface chemical groups were recorded on a Fourier transform infrared spectroscopy (FTIR, Nicolet iS50, ThermoFisher Scientific, MA, USA) using the attenuated total reflection (ATR) method in a range of 4000–400 cm^−1^. The compression test was performed on a universal testing machine (MST Insight 30, Minneapolis, MN, USA) with a compression rate of 2 mm/min from 0 to 80% strain. Thermal conductivity was measured by a thermal constant analyzer (TC3000E XIATECH, Xian, China), using the transient hot-wire method at room temperature. The thermographic images were obtained by an infrared thermal camera (FLIR T540, Wilsonville, OR, USA). TG-DSC analysis was conducted with a thermal analyzer (NETZSCH STA 4493F, Selb, Germany), with a heating rate of 10 °C/min from room temperature to 800 °C under air atmosphere. The gross calorific value (GCV) was obtained by an oxygen bomb calorimeter (C3000, IKA, Staufen, Germany). More than 0.3 g of sample was placed in the crucible and the vessel was pressurized up to 3 MPa using oxygen gas. The combustion behaviors were investigated by a cone calorimeter device (VOUCH 6810) according to the standard method ISO 5660: 2015. Samples with a size of 100 mm × 100 mm × 10 mm were tested under a heat flux of 50 kW/m^2^.

## Figures and Tables

**Table 1:** The temperatures at the maximum decomposition rates (T_max_), the maximum thermal decomposition rates (dW/dT) and the residues of the pure PVA aerogel, PVA-MMT-AP_0_ and PVA-MMT-AP_1.0_.

Samples	T_max_ (°C)		dW/dT (%/°C)		Residue (%)
	Stage II	Stage III	Stage II	Stage III	
PVA aerogel	329	476	0.58	0.62	4
PVA-MMT-AP_0_	272	435	0.40	0.16	47
PVA-MMT-AP_1.0_	292	500	0.23	0.26	43

**Table 2:** The combustion parameters of the PVA aerogel, PVA-MMT-AP_0_ and PVA-MMT-AP_1.0_.

Samples	TTI (s)	PHRR (kW/m^2^)	TTPHRR (s)	THR (MJ/m^2^)	FIGRA (kW/m^2^/s)	TSR (m^2^/m^2^)	Residue (%)
PVA aerogel	~2	562.8	72.1	46.2	7.8	746.5	0
PVA-MMT-AP_0_	~2	150.5	27.1	9.8	5.6	93.4	42.5
PVA-MMT-AP_1.0_	~2	130.6	28.0	12.0	4.7	96.5	39.1

## Data Availability

Not applicable.

## Supplementary Materials

The following supporting information can be downloaded at: https://www.mdpi.com/article/10.3390/gels8100654/s1, Figure S1: COMSOL simulation of the thermal insulation performance of the aerogel composites in the axial and radial directions [58,59]; Table S1: Skeletal density (*ρ_s_*), bulk density (*ρ*_b_) and radial shrinkage (S) of the PVA aerogel and PVA-MMT-AP_x_; Table S2: Pore parameters of the PVA aerogel and PVA-MMT-AP_x_. The pore volume (V_pore_), specific surface area (S_BET_) and average pore size (D_pore_) are determined from N_2_ adsorption analysis; the total pore volume (Vtotal) and porosity are calculated manually; Table S3: Pore parameters of PVA-MMT-AP1.0 determined from mercury porosimetry, including the pore volume (V_pore_), specific surface area (S_BET_), average pore size (D_pore_), bulk density (*ρ*_b_), skeletal density (*ρ*_s_) and porosity; Table S4: Solid thermal conductivity and interfacial thermal resistance R_k_ values of the individual components of the aerogel composite [44,53,60]; Video S1: Heat transfer along the axial direction; Video S2: Heat transfer along the radial direction.
