# Supplementary material for: Aramid Pulp Reinforced Clay Aerogel Composites: Mechanical, Thermal and Combustion Behavior
Source: Gels. 2022 Oct 14;8(10):654. doi: 10.3390/gels8100654 (PMC9601384; doi:10.3390/gels8100654)
Supplement: Supplementary file 1 [file gels-08-00654-s001.zip › Supplymentary Material.pdf]

Supplementary Material
Aramid Pulp Reinforced Clay Aerogel Composites: Mechanical, Thermal and Combustion Behavior

Xiaowu Wang [1], Yang Wang [1], Mengtian Sun [1], Guichao Wang [1], Qiong Liu [1], Ming Li [1], Yury M. Shulga [2,3] and Zhi Li [1,*]

[1] School of Resource and Safety Engineering, Central South University, Changsha 410083, China
[2] Institute of Problems of Chemical Physics, Russian Academy of Sciences, 142432 Chernogolovka, Russia
[3] National University of Science and Technology MISIS, Leninsky pr. 4, 119049 Moscow, Russia
* Correspondence: lizhi89@csu.edu.cn

Table S1. Skeletal density (ρ_s), bulk density (ρ_b) and radial shrinkage (*S*) of the PVA aerogel and PVA-MMT-AP_x.

**Sample**	**Skeletal density (g/cm^3)**	**Bulk density (g/cm^3)**	**Radial shrinkage (%)**
PVA aerogel	1.300	0.118 ± 0.001	8.69 ± 0.43
PVA-MMT-AP_0	1.733	0.067 ± <0.001	2.35 ± 0.55
PVA-MMT-$AP_{0.5}$	1.717	0.071 ± 0.001	2.11 ± 1.02
PVA-MMT-$AP_{1.0}$	1.703	0.070 ± <0.001	3.23 ± 0.34
PVA-MMT-$AP_{2.0}$	1.677	0.073 ± 0.002	1.80 ± 1.13

Table S2. Pore parameters of the PVA aerogel and PVA-MMT-AP_x. The pore volume (V_{pore}), specific surface area (S_{BET}) and average pore size (D_{pore}) are determined from N_2 adsorption analysis; the total pore volume (V_{total}) and porosity are calculated manually.

Sample	V_{pore} (cm^3/g) [a]	S_{BET} (m^2/g) [b]	D_{pore} (nm) [c]	V_{total} (cm^3/g) [d]	Porosity (%)
PVA aerogel	0.048	5.089	25.69	7.71	90.93 ± 0.10
PVA-MMT-AP_0	0.065	7.727	21.68	14.35	96.16 ± 0.05
PVA-MMT-$AP_{0.5}$	-	-	-	-	95.88 ± 0.06
PVA-MMT-$AP_{1.0}$	0.101	9.686	33.16	13.70	95.88 ± 0.03
PVA-MMT-$AP_{2.0}$	0.067	7.969	23.40	13.10	95.65 ± 0.10

[a, c] V_{pore} and D_{pore} are calculated from nitrogen adsorption isotherms using the BJH (Barrett-Joyner-Halenda) method.

[b] S_{BET} is calculated from nitrogen adsorption-desorption isotherms in the relative pressure range from 0.027 to 0.253 using the BET (Brunauer-Emmett-Teller) method.

[d] V_{total} is calculated by V_{total} = $(1/\rho_b - 1/\rho_s)$, where ρ_b and ρ_s are the bulk density and skeletal density, respectively.

Table S3. Pore parameters of PVA-MMT-$AP_{1.0}$ determined from mercury porosimetry, including the pore volume (V_{pore}), specific surface area (S_{BET}), average pore size (D_{pore}), bulk density (ρ_b), skeletal density (ρ_s) and porosity.

Sample	V_{pore} (cm^3/g)	S_{BET} (m^2/g)	D_{pore} (nm)	ρ_b (g/cm^3)	ρ_s (g/cm^3)	Porosity (%)
PVA-MMT-$AP_{1.0}$	12.33	157.15	313.83	0.077	1.508	94.89

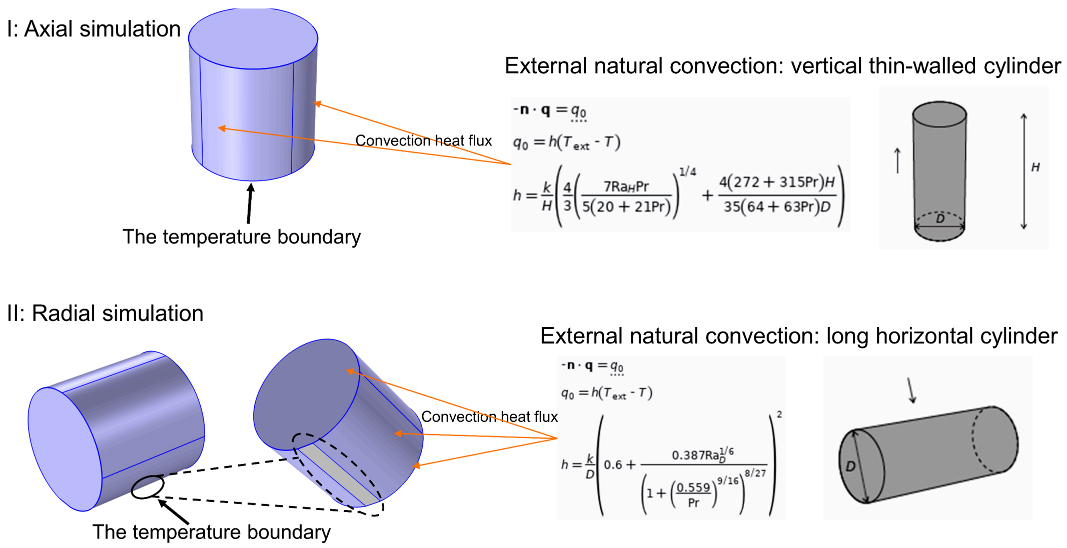

Figure S1. COMSOL simulation of the thermal insulation performance of the aerogel composites in the axial and radial directions.

The temperature boundary and the convection heat flux are set as shown in Fig. S1, and the equations of convection heat flux are listed on the right. Note that, a small enough plane was dug out to be the temperature boundary surface in the radial simulation aiming to define the temperature boundary. The parameters including the thermal conductivity and density are kept consistent with PVA-MMT-$AP_{1.0}$. The specific heat capacity was 1131 J K^{-1} g^{-1}, which is a weighted average of the specific heat capacity of the individual components of the aerogel composite. Specifically, the specific heat capacity of MMT, PVA and AP are obtained from the literature reported [58,59] and the Dupont aramid product manual.

Discussion S1: estimation of the gas conduction

The gaseous thermal conductivity in free space λ_{g0} can be approximated using a fourth-order polynomial of temperature as Eq. (.

$$\begin{aligned}\lambda_{g0} &= 1.6241\times10^{3} + 8.4798\times10^{-5}T + 2.8587\times10^{-9}T^{2} \\ &-3.7581\times10^{-11}T^{3} + 1.6705\times10^{-14}T^{4}\end{aligned} \tag{1}$$

β can be calculated by Eq. (2).

$$\beta = \frac{5\pi}{32}\frac{2-\alpha}{\alpha}\frac{9\gamma-5}{\gamma+1} \tag{2}$$

where α is the accommodation coefficient, γ is the specific heat ratio. For air in aerogel, $\beta \approx 2$.

K_n can be estimated as Eq. (3).

$$K_n = \frac{l_m}{\delta} \tag{3}$$

where l_m represents the mean free path of a gas molecule, δ is the characteristic system size, usually can be taken as the average pore size of the porous aerogel. Herein, δ is 313.83 nm obtained from the mercury porosimetry. l_m can be estimated from Eq. (4).

$$l_m = \frac{k_B T}{\sqrt{2}\pi d_g^2 P} \tag{4}$$

where k_B is the Boltzmann constant, d_g is the diameter of the air molecule, T is the temperature, and P is the pressure.

Discussion S2: estimation of the solid conduction

The solid conduction of the cell wall can be estimated by a weighted average of the solid conduction values λ_{sol}^{*} of the individual components of the aerogel composite. For a bulk material with a composition identical to the aerogel composite (45.5% wt% MMT, 45.5% wt% PVA and 9.1 wt% AP), the overall conduction is estimated to be ~91 mW m^{-1} K^{-1} based on the individual solid conduction provided in Table S4. However, the solid conduction of this nanosized material is greatly reduced due to the interfacial phonon scattering. The effective solid conductivity λ_{sol}^{*} can be estimated by

$$\lambda_{sol}^{*} = \frac{\lambda_{sol}}{1+\lambda_{sol}\frac{R_k}{d}} \quad (5)$$

where R_k is the Kapitza resistance and d is the particle size. The weighted average of the solid conduction (λ_s) of the aerogel composite is about 43 mW·m^{-1}·K^{-1}.

Table S4. Solid thermal conductivity and interfacial thermal resistance R_k values of the individual components of the aerogel composite.

**Components**	λ_{sol} **(W·m⁻¹·K⁻¹)**	R_k **×10⁻⁸ (m²·K·W⁻¹)**	d **(nm)**	λ^*_{sol} **(W·m⁻¹·K⁻¹)**
MMT[44]	0.04	0.7	25	0.040
PVA[53]	0.15-0.3	6.7-13.3	10	~0.050
AP[60]	0.05	10	4	0.022
